# The pathological landscape of tumor evolution: a paradigm shift from morphology to functional networks

**DOI:** 10.3389/fonc.2026.1842639

**Published:** 2026-04-28

**Authors:** Ying Yuan, Yiran Sun, Ying Ma, Hong Liu, Kangrong Sun

**Affiliations:** 1 Department of Pathology, Chongqing Wulong People’s Hospital, Chongqing, China; 2 School of Clinical Medicine, North Sichuan Medical College, Nanchong, Sichuan, China; 3 Nephrology Department, Chongqing Wulong People’s Hospital, Chongqing, China

**Keywords:** cancer-associated fibroblasts, spatial pathology, therapeutic resistance, tumor heterogeneity, tumor microenvironment

## Abstract

Tumor pathology is undergoing a profound transformation, shifting from pure morphological description toward multidimensional functional network analysis. This Mini Review focuses on the tumor microenvironment (TME) as a central concept driving tumor initiation, progression, and therapeutic resistance. We first outline the limitations of traditional pathological classification and elaborate on how the dynamic co-evolution of cellular components (such as cancer-associated fibroblasts (CAFs) and immune cells) along with the extracellular matrix (ECM) constitutes a functional unit. Key controversies are discussed, including the translational hurdles of TME-directed therapies and the challenge of spatiotemporally assessing tumor heterogeneity. We further identify critical research gaps, particularly the mechanistic understanding of the tumor-host interface across scales. Finally, we envision that the integration of artificial intelligence–driven spatial pathology, single-cell multi-omics, and *in vivo* imaging will usher in a new era of “functional pathology,” merging morphology, molecular profiling, and dynamic insights.

## 1 Introduction

Tumor pathology serves as a critical bridge between basic science and clinical oncology, with the core missions of accurate diagnosis, treatment guidance, and prognostic prediction. For over a century, tumor classification, grading, and staging systems (such as the TNM system), grounded in microscopic morphology, have formed the cornerstone of clinical decision-making. However, with the rapid advancement of molecular biology, immunology, and single-cell sequencing technologies, it has become increasingly clear that tumors are not isolated entities of monoclonal cancer cell proliferation, but rather highly complex, dynamically evolving “organ-like” ecosystems (1). This ecosystem comprises cancer cells, diverse infiltrating immune cells, cancer-associated fibroblasts (CAFs), endothelial cells, and the extracellular matrix (ECM) collectively constructed and modified by these components, which is collectively known as the tumor microenvironment (TME).

Although traditional pathological assessments have recognized certain features of the TME (e.g., lymphocytic infiltration), these have often been regarded as background or qualitative descriptors rather than quantifiable, targetable functional systems (2). This limitation is particularly evident in the current era of precision medicine, where tumor biological behaviors, such as local invasion, distant metastasis, and responses to chemotherapy, targeted therapy, and immunotherapy, are profoundly regulated by the intricate signaling networks within the TME. Consequently, expanding the pathological perspective from a cancer cell–centric “cellular pathology” to a “microenvironment pathology” encompassing all TME components and their interactions has become an urgent priority.

This Mini Review explores this paradigm shift, focusing on recent conceptual advances in understanding the TME as a central driver of tumor evolution. We first discuss key components of the TME and their functional heterogeneity, then analyze current controversies and research gaps regarding therapeutic targeting and heterogeneity assessment, and finally envision how future technological integration will reshape the practice of tumor pathology.

## 2 The tumor microenvironment: the new center of pathology

### 2.1 Beyond stroma: the functional duality of cancer-associated fibroblasts

CAFs are the most abundant stromal cells in the TME and have long been viewed merely as the “scaffold” of the tumor interstitium. However, single-cell transcriptomic and lineage-tracing studies have revealed that CAFs constitute a highly heterogeneous cell population with distinct, even opposing, functions. In early tumorigenesis, certain CAF subsets (e.g., myofibroblasts expressing α-smooth muscle actin, α-SMA) promote cancer cell proliferation, invasion, and epithelial-mesenchymal transition (EMT) by secreting abundant ECM components (such as collagen and fibronectin) and growth factors (e.g., hepatocyte growth factor, HGF; transforming growth factor-β, TGF-β) (3). In pathological sections, this is often manifested as dense, bundle-like “desmoplastic reactions,” a hallmark of many solid cancers (e.g., pancreatic and breast cancer) and associated with poor prognosis (4).

However, recent studies have also identified CAF subsets with tumor-suppressive functions. For instance, in pancreatic cancer, CAFs expressing major histocompatibility complex class II (MHC-II) are thought to participate in antigen presentation, while CAF subsets with low α-SMA expression correlate with longer patient survival (5). This functional “duality” poses a major challenge for CAF-targeted therapies: non-selective depletion of CAFs may inadvertently accelerate tumor progression by also eliminating their suppressive subsets (6). Thus, from a pathological perspective, a future challenge lies in accurately identifying and quantifying these functionally distinct CAF subsets *in situ* using multiplex immunohistochemistry (mIHC) or spatial transcriptomics, thereby providing a pathological basis for developing subset-specific targeting strategies.

### 2.2 The immune microenvironment: from the “hot” versus “cold” dichotomy to spatiotemporal dynamic networks

Assessment of the tumor immune microenvironment, particularly the density of CD8+ T cells, has become a critical biomarker for predicting response to immune checkpoint inhibitors (ICIs). The classic classification of “hot” (highly infiltrated by T cells) versus “cold” (sparsely infiltrated by T cells) tumors provides a preliminary framework for clinical decision-making (7). However, this binary classification is overly simplistic. Pathological analyses reveal that even in “hot” tumors, T cells may be functionally exhausted (characterized by high expression of programmed cell death protein 1, PD-1, and T cell immunoglobulin and mucin domain-containing protein 3, Tim-3) (8) or physically constrained by immunosuppressive cells (e.g., regulatory T cells, Tregs; myeloid-derived suppressor cells, MDSCs; tumor-associated macrophages, TAMs) and the chemical gradients they create (9).

Spatial pathology technologies, such as imaging mass cytometry (IMC) and multiplexed ion beam imaging (MIBI), allow simultaneous analysis of dozens of markers at single-cell resolution and precise reconstruction of spatial relationships among cells. These studies have unveiled complex architectures at the tumor–immune interface, exemplified by the identification of distinct immune neighborhoods associated with clinical outcomes (10) and the discovery of organized cellular communities driving immunosuppression (11). Beyond spatial organization, the functional state of immune cells is critically shaped by intrinsic regulatory molecules. For instance, the butyrophilin family member BTN2A2 has been shown to inhibit T cell activation and proliferation, promote the induction of regulatory T cells (Tregs), and skew macrophage polarization toward an anti-inflammatory M2 phenotype in autoimmune disease models (ref), mechanisms that may also be relevant in the tumor immune microenvironment, particularly in shaping immunosuppressive niche (12).For example, the “immune-excluded” phenotype (T cells aggregating only in the stroma surrounding tumor nests) and the “immune-desert” phenotype (absence of T cells throughout the tissue) exhibit distinct pathological features and therapeutic response profiles (13). Furthermore, the discovery of tertiary lymphoid structures (TLSs), which are organized aggregates of B cells, T cells, and dendritic cells formed in non-lymphoid tissues, adds a new dimension to tumor immunopathology (14). Mature TLSs are often associated with favorable ICI efficacy and prognosis, and are regarded as local “command centers” orchestrating antitumor immune responses. Therefore, future pathological diagnostics should move beyond merely reporting immune cell density to systematically characterize their spatial distribution, functional status, and structural organization, generating a “spatial map” of tumor–immune interactions.

## 3 Current controversies and research gaps

### 3.1 Translational challenges: TME-targeted therapeutic strategies—hope and hurdles

Given the central role of the TME in tumor progression, targeting the TME has become a frontier in oncotherapy. However, several promising strategies have encountered bottlenecks in clinical translation, reflecting an incomplete understanding of TME complexity and plasticity.

Targeting CAFs: As noted, the functional heterogeneity and plasticity of CAFs represent major obstacles. Drugs targeting key signaling pathways involved in CAF activation (e.g., the Hedgehog pathway), such as vismodegib, failed to improve survival in pancreatic cancer patients and may have even accelerated tumor invasion by weakening the ECM barrier (3). This lesson underscores the necessity of subtype-specific and spatiotemporally selective approaches for TME-directed therapies.

Immunotherapy: Although ICIs have achieved success in multiple cancer types, primary or acquired resistance remains a common challenge. Pathological studies indicate that resistance mechanisms are multifaceted, including intrinsic defects in antigen presentation machinery within cancer cells (e.g., B2M mutations) (8), persistent metabolic stress within the TME (e.g., hypoxia, acidosis), and reorganization of immunosuppressive cells. Simply increasing T cell infiltration (converting “cold” tumors to “hot”) does not always translate into efficacy, as infiltrating T cells may rapidly become functionally exhausted. Therefore, the rationale for combination strategies (e.g., ICIs with antiangiogenic agents or with agents targeting TAMs/MDSCs) lies in simultaneously relieving multiple layers of immunosuppression. However, the optimal combination, sequence, and pathological criteria for selecting suitable patient populations remain open questions. In addition to resistance mechanisms originating from the TME, the clinical management of immune-related adverse events (irAEs) itself may influence treatment outcomes and potentially reshape the immune microenvironment. For instance, the use of high-dose corticosteroids to manage severe irAEs, a common reason for immunotherapy discontinuation, may modulate immune cell function and TME composition, although the long-term impact on tumor control remains incompletely understood (15). Understanding how such therapeutic interventions intersect with TME dynamics represents an emerging area at the interface of clinical oncology and functional pathology.

### 3.2 Research gap: spatiotemporal dynamics and mechanisms of tumor heterogeneity

Tumor heterogeneity manifests not only across patients (inter-tumor heterogeneity) but also within a single tumor (spatial heterogeneity) and across different stages of disease progression (temporal heterogeneity). Currently, clinical practice still relies on a single biopsy to represent the entire tumor, inevitably leading to sampling bias (16). Nevertheless, significant knowledge gaps remain in two critical areas. First, the functional correlates of spatial heterogeneity remain poorly defined; specifically, it is unclear how regional variations in mutational landscapes, phenotypic traits, and TME composition are mechanistically linked, and whether distinct spatial “niches” directly govern clonal selection, invasion, or immune escape. Second, the drivers of temporal heterogeneity present a major challenge.

Drivers of temporal heterogeneity: Under therapeutic pressure (e.g., chemotherapy, targeted therapy), how do cancer cells and the TME co-evolve? How does therapy-induced senescence-associated secretory phenotype (SASP) reshape the TME and potentially contribute to relapse or metastasis (17)? Currently, there is a lack of non-invasive tools for real-time, continuous monitoring of such spatiotemporal evolution *in vivo*, along with matched high-resolution pathological validation. In parallel, the clinical diagnosis and risk stratification of tumors often rely on conventional serum biomarkers or single-site biopsies, which may not reflect underlying tumor microenvironment heterogeneity. For example, cases have been reported where patients with normal total prostate-specific antigen (PSA) levels but elevated free PSA density (fPSAD) were ultimately diagnosed with prostate cancer (18), highlighting a diagnostic gap that may be explained by regional variations in tumor-stroma interactions or immune infiltration patterns. This underscores the need to integrate TME-based spatial biomarkers into routine diagnostic workflows.

## 4 Discussion: toward the era of functional pathology

**Figure 1:**
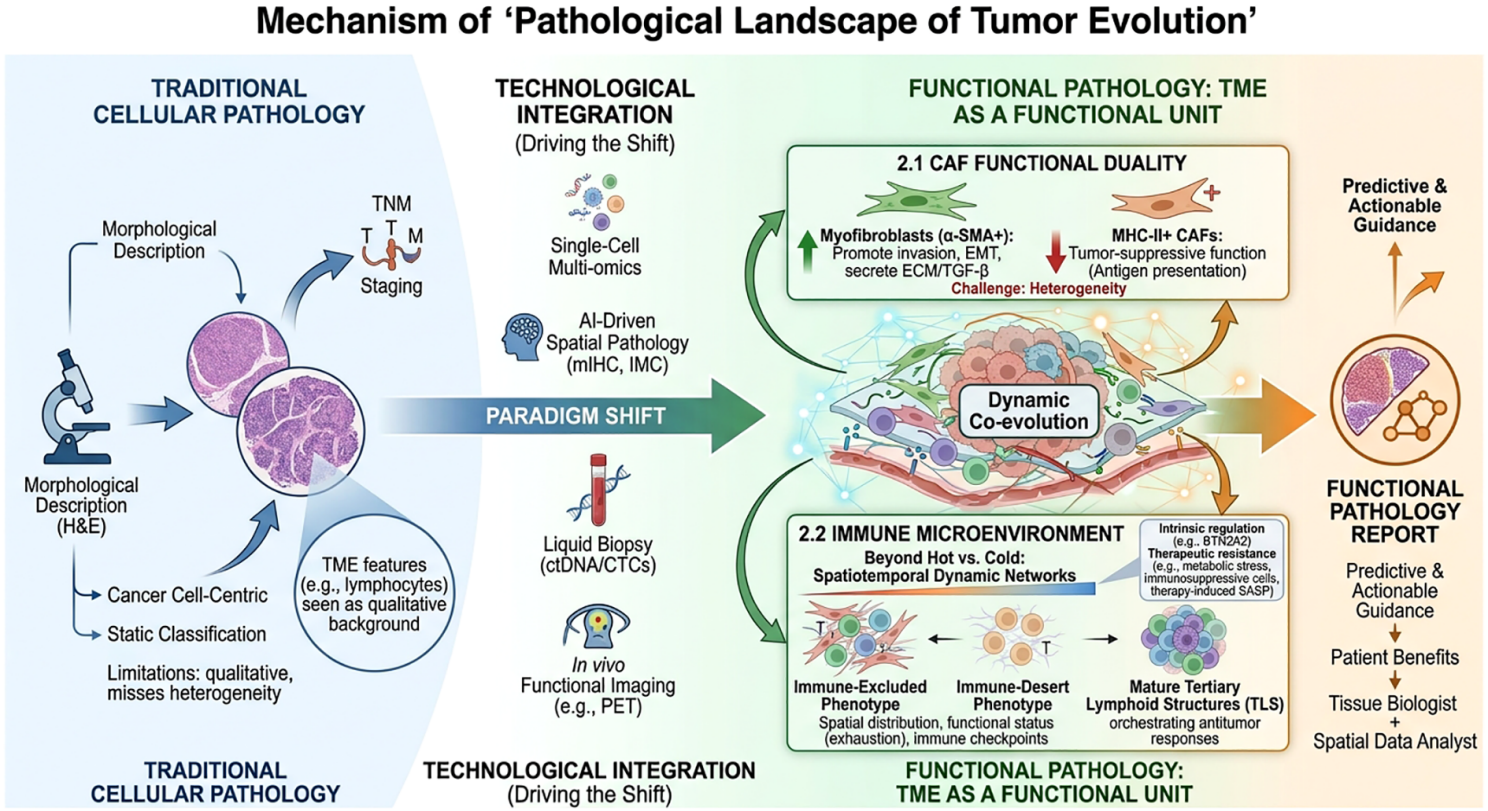
Schematic overview of the paradigm shift from traditional cellular pathology to functional pathology in tumor evolution. Traditional pathology, centered on morphological description and cancer cell-centric static classification, is limited by its qualitative nature and inability to capture heterogeneity. A paradigm shift is driven by the integration of advanced technologies, including single-cell multi-omics, artificial intelligence–driven spatial pathology (e.g., multiplex immunohistochemistry, imaging mass cytometry), liquid biopsy, and *in vivo* functional imaging. This transition establishes a functional pathology framework in which the tumor microenvironment (TME) is recognized as a dynamic functional unit. Key features include the functional duality of cancer-associated fibroblasts (CAFs), spatiotemporal immune networks beyond the “hot vs. cold” dichotomy, and mechanisms driving therapeutic resistance. Ultimately, this approach aims to provide predictive and actionable guidance in precision oncology, positioning pathologists as tissue biologists and spatial data analysts.

Addressing these challenges and gaps will inevitably lead tumor pathology toward deeper integration and quantification. We propose that the convergence of several key directions will define the next generation of tumor pathology (**Figure 1**).

Artificial Intelligence–Driven Spatial Pathology: AI algorithms, particularly deep learning-based image analysis, are already capable of extracting prognostic information from routine hematoxylin and eosin (H&E)-stained sections that surpasses human visual perception (19). In the future, AI will seamlessly integrate multimodal data from mIHC, spatial transcriptomics, and spatial proteomics to construct holistic pathological maps that encompass cell identity, functional status, spatial neighborhoods, and molecular pathways. This will elevate the pathology report from a statement such as “abundant CD8+ T cell infiltration” to a high-dimensional diagnostic description like “presence of a functionally exhausted CD8+ T cell zone at the invasive front, surrounded by PD-L1+ TAMs, forming immunosuppressive synapses with CAF subset A.”

From 2D Cross-Section to 3D Spatiotemporal Dynamics: A critical advancement on the horizon is the transition from analyzing single 2D tissue sections to reconstructing 3D tumor ecosystems. As noted in this review, 2D analysis, while foundational, offers an inherently incomplete view of the complex cellular neighborhoods and receptor-ligand interactions that govern tumor evolution. Recent proof-of-principle studies, such as the work by Pentimalli et al., have elegantly demonstrated the power of combining 3D spatial transcriptomics with extracellular matrix (ECM) imaging (for instance, second harmonic generation) in clinical samples (20). Their findings underscore that 3D neighborhoods are essential for restoring spatial continuity, revealing, for instance, that seemingly disconnected T cell niches in 2D are actually interconnected bridges in 3D volumetric space. More importantly, this 3D perspective is crucial for precisely mapping the functional duality of CAFs mentioned earlier in this review; it allows for the identification of specific CAF subsets (e.g., IGF1+ activated fibroblasts or TIMP1+ reticular fibroblasts) within their native 3D ECM compartments, thereby linking gene expression to local matrix remodeling and immune exclusion with unprecedented fidelity. This shift toward 3D functional pathology will be indispensable for accurately targeting microenvironment-directed therapies and overcoming the sampling bias inherent in 2D biopsies.

Complementarity of Liquid Biopsy and Tissue Pathology: Detection of circulating tumor DNA (ctDNA) and circulating tumor cells (CTCs) provides a global molecular profile and dynamic evolutionary landscape of the tumor. Integrating these “liquid biopsy” data with spatial information from tissue pathology holds promise for generating a panoramic view of tumor heterogeneity (21). For example, ctDNA can signal the emergence of a resistant clone, while tissue pathology can localize the niche in which that clone resides, revealing the TME conditions that support its outgrowth.

Integration of Functional Imaging and Pathology: Advanced imaging modalities, such as positron emission tomography (PET), are increasingly developing tracers specific to TME components (e.g., fibroblast activation protein, FAP; PD-L1). Correlating and validating such *in vivo* functional imaging data with the “gold standard” of pathology will enable non-invasive, real-time tracking of TME dynamics during treatment, facilitating “repeated, spatiotemporally dynamic pathological assessment (22).”

In summary, we stand at a crossroads in the evolution of tumor pathology. Traditional morphological pathology will remain the cornerstone of diagnosis, but its meaning is being profoundly enriched. The future pathologist will need to function as a “tissue biologist” and a “spatial data analyst,” capable of interpreting the intricate functional network woven by cancer cells, immune cells, stromal cells, and their secreted products. This transition toward “functional pathology” will not only deepen our understanding of tumor biology but also provide more predictive and actionable guidance for precision oncology, ultimately benefiting patients.
